# Dietary Changes during the COVID-19 Pandemic: A Longitudinal Study Using Objective Sequential Diet Records from an Electronic Purchase System in a Workplace Cafeteria in Japan

**DOI:** 10.3390/nu13051606

**Published:** 2021-05-11

**Authors:** Mieko Nakamura, Yoshiro Shirai, Masae Sakuma

**Affiliations:** 1 Department of Community Health and Preventive Medicine, Hamamatsu University School of Medicine, Hamamatsu 431-3192, Japan; 2 Department of Food and Nutritional Environment, Kinjo Gakuin University, Nagoya 463-8521, Japan; y-shirai@kinjo-u.ac.jp; 3 Department of Food and Health Sciences, International College of Arts and Sciences, Fukuoka Women’s University, Fukuoka 813-8529, Japan; m-sakuma@fwu.ac.jp

**Keywords:** COVID-19, real-world data, occupational health, dietary habits, vegetable intake, seasonal variation

## Abstract

As a result of the coronavirus disease 2019 (COVID-19) pandemic-related restrictions, food systems have undergone unprecedented changes, with the potential to affect dietary behavior. We aimed to investigate workers’ dietary changes resulting from the introduction of regulations to combat COVID-19 in a Japanese factory cafeteria. Objective data on daytime dietary intake were automatically collected from electronic purchase system records. The dataset included the weekly data of 890 men from 1 July 2019 to 30 September 2020. The cafeteria regulations came into effect on 10 April 2020; in this context, the purchase of dishes and estimated dietary intake were monitored. The number of cafeteria visits decreased slightly after the introduction of the regulations. The purchase of main and side dishes also decreased, but the purchase of grain dishes was less affected. When compared with summer 2019 (pre-pandemic, no regulations: 1 July to 29 September 2019), in summer 2020 (during the pandemic and with regulations: 29 June to 30 September 2020), the estimated mean grain, meat, fish, and total energy intake was stable; however, vegetable intake decreased by 11%. As the COVID-19 pandemic continues, workplace cafeteria regulations need to be monitored to avoid unfavorable dietary changes in employees.

## 1. Introduction

The coronavirus disease 2019 (COVID-19) pandemic and related restrictions have led to widespread changes; this also applies to lifestyles, including eating habits [1,2,3,4,5,6,7,8,9]. So far, as a result of the pandemic, Japan has twice experienced a state of emergency. The first state of emergency was declared in seven prefectures (the Tokyo metropolitan area, Osaka, Hyogo, and Fukuoka) on 7 April 2020 [10,11]. On 16 April, the target area was enlarged to include all 47 prefectures [10,11]. The state of emergency ended on 25 May [10,11]. The number of COVID-19-positive cases in Japan was 708 on 10 April 2020, at the peak of the first wave; 1595 on 7 August 2020, at the peak of the second wave; and 7844 on 8 January 2021, at the peak of the third wave [12].

Against this background, COVID-19-related regulations, based on the concept of the “Three Cs”—avoiding closed spaces with poor ventilation; avoiding crowded places with many people nearby; and avoiding close-contact settings, such as close-range conversations [13,14]—were introduced in many Japanese workplaces. These have been particularly relevant to workplace cafeterias.

Pandemic-related restrictions can affect dietary behavior across generations and countries. For example, a citywide lockdown would directly affect grocery shopping and eating out. Further, remote work and studying would affect mealtimes as well as the amount and type of food consumed. In fact, COVID-19-related studies have reported dietary behavior changes in children with obesity in Italy [1]; adolescents in Croatia [2]; adults in the USA [3], Denmark [4], France [5], Italy [6], Lithuania [7] and China [8]; and older adults in the Netherlands [9].

Even without citywide lockdowns and remote work, dietary habits can be affected by regulations in workplace cafeterias. Recently, some workplace cafeterias in Japan have introduced an electronic purchase system. The purchase records and recipe data obtained from such a system can help in automatically and objectively monitoring the dietary intake of each employee, without reliance on memory.

Therefore, we aimed to investigate the dietary changes that occurred during the implementation of several regulations based on the Three Cs in a workplace cafeteria in Japan, where the number of COVID-19 cases is still lower than in Western countries [15]. To the best of the authors’ knowledge, this study is the first to examine this issue based on objective daily sequential dietary data from an electronic purchase system in a workplace cafeteria. This study highlights the fact that the implementation of several regulations has had an unfavorable effect on dietary intake.

## 2. Materials and Methods

### 2.1. Dataset

This study used the dataset of an ongoing longitudinal study investigating the association between the intake of functional foods and cardiometabolic health at an automobile manufacturing factory in Aichi Prefecture, Japan (Shirai et al., submitted). In the study in question, each employee’s daytime dietary intake is automatically collected from the records of an electronic purchase system in the workplace cafeteria. The dataset includes the daytime dietary intake of 890 men (840 manual workers, 38 technical workers, and 12 desk workers) from 1 July 2019 (Week 1) to 30 September 2020 (Week 66), but not from Weeks 7, 27, 45, and 59, during which the factory was closed owing to the work schedule.

The participants were informed about the study protocol and their ability to opt out. No one refused to join the study. The study was approved by the ethics committees of Toyota Motor Corporation, Hamamatsu University School of Medicine, and Sugiyama Jogakuen University.

### 2.2. Purchase and Dietary Variables Included in the Dataset

The outcome variables were the number of dishes purchased and estimated food and nutrient intake, calculated using the purchase data.

In the workplace cafeteria, employees choose plates from the display case and place them on their trays. After the meal and at the time of payment, the electronic information held in an integrated circuit chip attached to each plate on the tray is recorded along with the employee identification card, and the number of dishes purchased is automatically obtained for each employee.

In this study, we counted the number of dishes purchased based on the typical dish classification (“syusyoku”, “syusai”, and “fukusai”) in the Japanese Food Guide Spinning Top [16,17]. Syusyoku refers to grain dishes, which mainly include boiled rice and sometimes bread, noodles, or pasta. Syusai refers to main dishes, which usually consist of meat or fish and sometimes egg or soybean. Fukusai refers to side dishes, which contain mainly vegetables. In addition, we counted the number of “healthy set meals” purchased; these are nutritionally well-balanced pre-set meals planned by a dietitian that usually contain syusyoku, syusai, and fukusai.

Then, estimated dietary intake was calculated based on the purchase data for each dish and recipe database. In other words, the daily purchase information of dishes (syusyoku, syusai, fukusai, and healthy set meals) was converted to recipe data. Recipe data comprise food data with some nutrient data (total energy, macronutrients, and salt), which were pre-calculated using the Standard Tables of Food Composition in Japan–2015 based on the Food Labeling Act [18]. Leftovers were not considered for estimation of dietary intake because of a lack of information.

Although the system recorded daily original purchase big data for 66 weeks, we were offered summarized data on a weekly basis. During the 66 weeks, there were no changes in the availability of fukusai, vegetables, and other dishes or foods or in prices. As for drinks, there were no records pertaining to free water and green tea; however, there were records pertaining to free catechin-rich green tea as a functional food. The workplace cafeteria in question does not sell any other drinks.

### 2.3. Cafeteria Regulations in Response to the COVID-19 Pandemic

The factory management introduced the use of different time slots in the workplace cafeteria on 10 April 2020. First, lunchtime was divided into two time slots for departments that have a 45-min lunch break and three time slots for departments that have a 60-min lunch break. Then, the lunchtime was divided into two time slots for all departments, and the time difference was set at 15 min (i.e., the early lunchtime started at 12:00 and the late lunchtime started at 12:15). Employees were divided into either the early or late group, and the group was appropriately changed in each department. Regulations other than the cafeteria’s different time slots were as follows: a standard time for lunch (i.e., the time from the beginning of choosing dishes from the display case to finishing eating and leaving was limited to 15 min in principle); leaving the cafeteria as soon as having finished eating; not sitting face-to-face with someone else and maintaining social distancing at the table; keeping quiet while eating; and maintaining social distancing and wearing a mask when lining up at the display case. In addition to the regulations for employees, the management ventilated the cafeteria and disinfected the tables every day.

### 2.4. Statistical Analysis

The number of times each dish was purchased and estimated dietary intake were divided by the number of visits to the workplace cafeteria. Then, the longitudinal data for 66 weeks were described from the perspective of time in weekly graphs. Means and standard deviations for estimated dietary intake were calculated, and differences in the means between the regulation-free period (from 1 July 2019 to 12 April 2020) and the period of regulation (from 13 April to 30 September 2020), and between summer 2019, when there were no regulations (from 1 July to 29 September 2019), and summer 2020, when regulations were in effect (from 29 June to 30 September 2020), were tested using the Student’s t-test. Then, the proportion of the estimated dietary intake during summer 2020 compared to summer 2019 was shown in graph form. IBM SPSS Statistics for Windows, Version 25.0 (IBM Corp., Armonk, NY, USA) was used for the analysis. *p*-values < 0.05 were considered statistically significant.

## 3. Results

**Figure 1:**
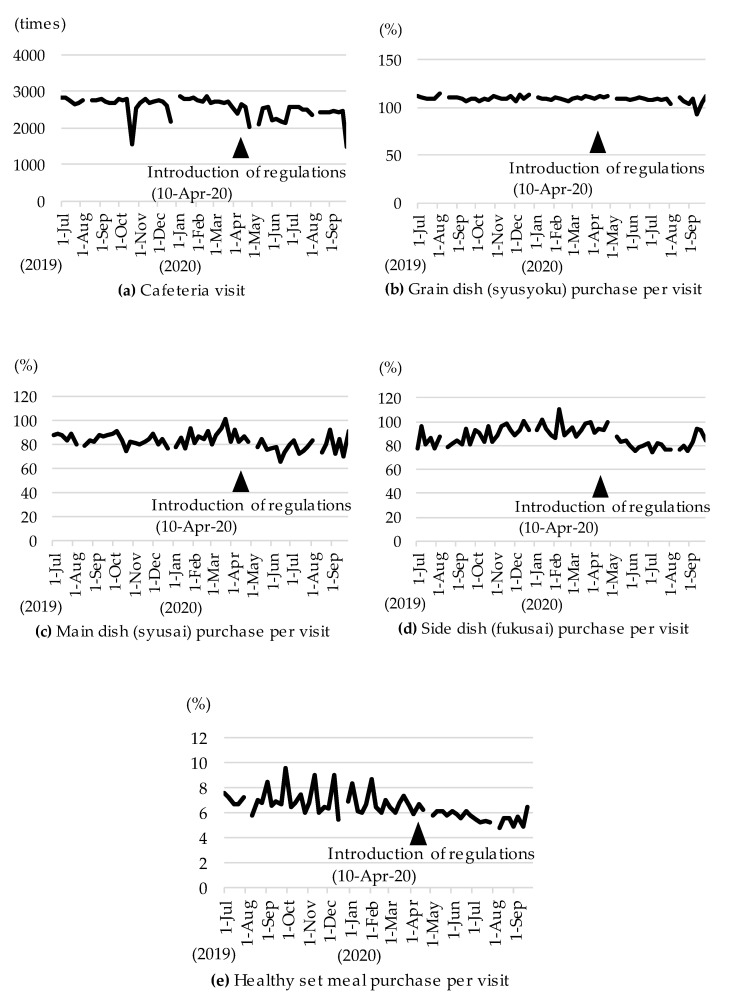
The number of visits to the workplace cafeteria and the purchase of dishes per cafeteria visit from 1 July 2019 to 30 September 2020. Pandemic regulations in the workplace cafeteria were implemented on 10 April 2020. (**a**) Cafeteria visit; (**b**) Grain dish (syusyoku) purchase per visit; (**c**) Main dish (syusai) purchase per visit; (**d**) Side dish (fukusai) purchase per visit; (**e**) Healthy set meal purchase per visit.

The workplace cafeteria visits and dishes purchased per cafeteria visit from 1 July 2019 (Week 1) to 30 September 2020 (Week 66) are shown in Figure 1. The number of visits to the workplace cafeteria decreased slightly after the introduction of the regulations (Figure 1a). Some steep declines were due to the absence of the factory’s work schedule. Taking into account the number of visits to the workplace cafeteria, the purchase of main and side dishes decreased after the regulations were introduced, but the purchase of grain dishes was less affected (Figure 1b–d). The purchase of healthy set meals also decreased after the introduction of the regulations (Figure 1e).

**Figure 2:**
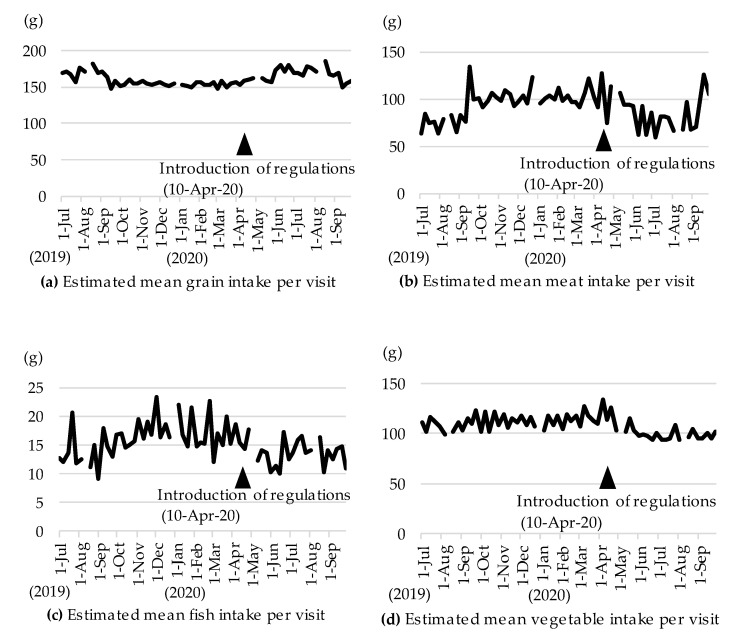
Estimated mean food intake per cafeteria visit from 1 July 2019 to 30 September 2020. Pandemic regulations in the workplace cafeteria were implemented on 10 April 2020. (**a**) Estimated mean grain intake per visit; (**b**) Estimated mean meat intake per visit; (**c**) Estimated mean fish intake per visit; (**d**) Estimated mean vegetable intake per visit.

The change in the estimated mean food intake per visit is shown in Figure 2. The estimated mean grain intake increased and the estimated mean meat, fish, and vegetable intake decreased after the introduction of the regulations. As for the trend throughout the study period, the estimated mean grain intake tended to be high from spring to summer and low from autumn to winter; in contrast, the estimated mean meat and fish intake tended to be low from spring to summer and high from autumn to winter.

The estimated mean food and nutrient intake was divided into two periods (Table 1): the regulation-free period and after the introduction of regulations. The estimated mean grain intake was significantly higher and the estimated mean fish, vegetable, total energy, protein, and carbohydrate intakes were significantly lower after the introduction of the regulations than in the regulation-free period.

When comparing the summer periods before and during the pandemic (i.e., summer 2019, no regulations vs. summer 2020, after the introduction of regulations), the estimated mean vegetable intake was significantly lower in summer 2020. However, the estimated mean intake of other foods and nutrients did not differ between the two periods. As seen in Figure 2 and Table 1, the low vegetable intake in summer 2020 could have been a result of the regulations, while the high grain intake and low fish intake could just have been a seasonal variation.

**Figure 3:**
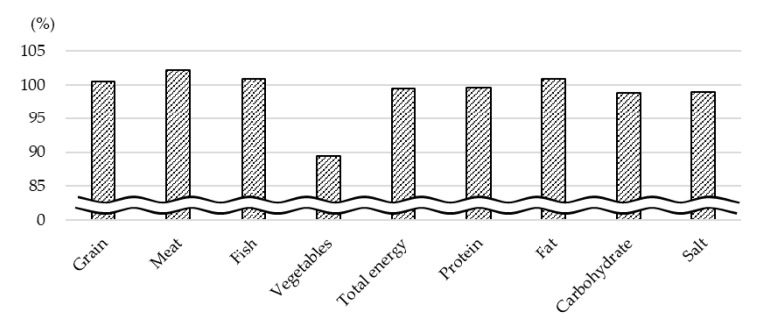
Proportion of the estimated mean food, total energy, and nutrient intake during summer 2020, when there were regulations, compared to summer 2019, when there were no regulations.

The intake percentages during summer 2020, when there were regulations, divided by those during summer 2019, when there were no regulations, are shown in Figure 3. Vegetable intake showed an 11% decrease, while the intake of other food and nutrients remained stable (−1 to +2%).

## 4. Discussion

In this longitudinal study using objective diet records taken from an electronic purchase system in a Japanese workplace cafeteria, it was found that there was an 11% decrease in vegetable intake during lunch in the summer of 2020, which was during the COVID-19 pandemic, compared to the pre-pandemic period of summer 2019, when the related regulations had not been implemented. This study also revealed that the high grain intake and low fish intake was just a seasonal variation [19,20], unconnected with the COVID-19 pandemic and the related regulations.

The decrease in vegetable intake was due to the reduced intake of side dishes. This could have happened as a result of employees’ behavior change in terms of less time to eat lunch and/or the time allowed for lining up to take dishes from the display case. In other words, employees prioritized eating grain and main dishes over side dishes when the mealtime was short, and limited their choosing time to maintain social distancing. Indeed, the factory management has sought to understand the changes employees may have made in response to the cafeteria regulations, discovering the following: (1) the frequency of using the cafeteria has decreased while that of bringing a lunch box has increased to eliminate waiting times; (2) the number of dishes purchased per person has slightly decreased because of the short cafeteria usage time; and (3) the amount of food consumed has slightly decreased to avoid weight gain, in consideration of the sedentary lifestyle brought about by the COVID-19-related restrictions.

Several studies have reported dietary change in adults because of the COVID-19 pandemic and related restrictions [3,4,5,6,7,8]. A decrease in whole fruit, vegetable, and lean protein intake, but an increase in sweet and snack and refined grain intake, were found in American adults within three months of the mandated quarantine [3]. Similarly, a decrease in fruit and vegetable intake, and an increase in fish intake, were observed in Danish adults during the COVID-19 lockdown [4]. In contrast, a large proportion of adults in Lithuania reported an increase in fruit and vegetable intake rather than a decrease, while a large proportion reported a decrease in red meat and fish intake rather than an increase one month after the beginning of the COVID-19 quarantine [7]. These studies indicate that the consumption of fruit and vegetables may be vulnerable to COVID-19 countermeasures.

The strength of this study is that dietary intake was assessed using objective sequential diet records collected by the electronic purchase system. As these records are obtained automatically, they are completely unaffected by recall bias. Using self-report to assess dietary intake may be influenced by the participants’ memories, and is usually difficult to evaluate quantitatively.

Nevertheless, this study does have several limitations. First, because this study was conducted at a single Japanese factory, the generalizability of the results is limited. However, as many factory workers in Japan usually take lunch in the workplace cafeteria, it is possible that a similar decrease in vegetable intake during the time of implementation of COVID-19 regulations may be observed in other factories as well. Second, the dietary records were limited to lunch. A previous study reported that Japanese middle-aged male workers consume 30–40% of their total energy at lunch during weekdays [21], and the dietary intake from lunch may have a considerable impact on total dietary intake. It is completely unclear whether the reduction in vegetable intake during lunch can be compensated for by breakfast and dinner. Third, the leftover food from lunch was not recorded in this study. A Japanese national survey revealed that the proportion of leftover food in cafeterias and restaurants was 3.6% [22], and another study reported that the proportions of leftovers were 6.7% for syusyoku, 4.9% for syusai, and 5.0% for fukusai among university students [23]. Thus, leftovers in the present study could have been around 3% but less than 5%, because the participants were more physically active than the previously studied university students. However, it is possible that there would be more leftover food during the COVID-19-related regulations because of the shortened lunchtime. Therefore, it is possible that the decrease in vegetable intake was larger than the amount recorded in this study. Fourth, the decrease in vegetable intake may happen if executives purchase more side dishes and consume more vegetables but were working remotely after the introduction of the regulations. However, only 1.4% of employees were desk workers who could potentially work remotely.

## 5. Conclusions

This study highlights that the short lunchtime and social distancing in the workplace cafeteria, implemented as countermeasures against the COVID-19 pandemic, can potentially have an unfavorable effect on workers’ dietary intake. Decreased vegetable intake is contrary to the target of Health Japan 21 (the second term), the national health promotion program [24]. As the COVID-19 pandemic continues, it is necessary to change long-term countermeasures in the workplace to promote health.

## Figures and Tables

**Table 1:** Estimated mean food, total energy, and nutrient intake pre-pandemic and after the introduction of regulations. Pandemic regulations in the workplace cafeteria were implemented on 10 April 2020. *p*-values were obtained by the Student’s *t*-test.

	No Regulations		After the Introduction of Regulations			2019 Summer without Regulations		2020 Summer with Regulations		
	1 July 2019 to 12 April 2020		13 April to 30 September 2020			1 July to 29 September		29 June to 30 September		
	Mean	SD	Mean	SD	*p*	Mean	SD	Mean	SD	*p*
Grain ^1^	158	8	167	9	<0.01	167	9	168	10	0.85
Meat ^1^	95	15	87	20	0.07	81	19	83	19	0.82
Fish ^1^	16	3	14	2	<0.01	14	3	14	2	0.91
Vegetables ^1^	112	8	101	8	<0.01	109	7	98	5	<0.01
Total energy ^2^	821	26	799	33	<0.01	800	22	796	33	0.74
Protein ^1^	30	1	29	1	<0.01	29	1	29	1	0.79
Fat ^1^	30	2	29	3	0.08	28	2	29	3	0.80
Carbohydrate ^1^	107	2	105	2	<0.01	106	3	105	2	0.22
Salt ^1^	4.0	0.1	4.0	0.2	0.31	4.1	0.1	4.0	0.2	0.37

^1^ g/visit, ^2^ kcal/visit, SD standard deviation.

## Data Availability

We are not able to share raw data because we have not explained the sharing of raw data in our ethical review or informed consent.
